# Genome Sequences of Akoni, Ashton, and Truong, *Podoviridae* Bacteriophages Isolated from Microbacterium foliorum

**DOI:** 10.1128/MRA.00516-21

**Published:** 2021-07-15

**Authors:** Fadia Fakhre, Raquel M. Gonzalez, Elisabeth K. Howells, Louis Otero, Kaili N. Pegoraro, Kobe C. Robichaux, Alexandra Rodier, Carter L. Sadowski, Victoria P. Carter, Amber D. Gray, Grace C. Klein, Catherine Lebosada, Chris M. Miklaszewski, Sydney N. Sutton, Michael A. Chase, Caitlyn N. Coleman, Brooke Corbett, Manoela O. Cunha, Marshall Daffner, Caitlyn J. Deam, Laura J. Deloso, Anthony M. DeSomma, Juan Pinera Gallardo, Mae E. Horne, Olivia Kanahan, Victor Lam, Ryan T. Morgan, Emilee M. Mustor, Migdalia Ricardo-Iglesias, Chloe J. Sartorio, Ava R. Sciacchitano, Alex W. Tvenstrup, Audrey R. Wood, Richard S. Pollenz

**Affiliations:** a Department of Cell Biology, Microbiology and Molecular Biology, University of South Florida, Tampa, Florida, USA; Queens College CUNY

## Abstract

Cluster EK2 Akoni, Ashton, and Truong are lytic *Podoviridae* actinobacteriophages that were isolated from soil in Florida using Microbacterium foliorum NRRL B-24224 as the host. The genomes are 54,307 bp, 54,560 bp, and 54,309 bp, respectively, and are 60% GC rich. Each genome contains a novel 13,464-bp gene that encompasses 25% of the genome.

## ANNOUNCEMENT

In 2017, the Science Education Alliance-Phage Hunters Advancing Genomics and Evolutionary Science (SEA-PHAGES) program (1) expanded the number of host bacteria beyond Microbacterium smegmatis in an effort to isolate evolutionarily diverse actinobacteriophages. Akoni, Ashton, and Truong were isolated directly from single moist soil samples taken from three Florida locations (Table 1) using Microbacterium foliorum NRRL B-24224 grown at 30°C on peptone-yeast calcium agar (PYCa). Genomic DNA was isolated after two rounds of plaque purification using the Wizard DNA cleanup kit (A7280; Promega). Genomic DNA was used to create sequencing libraries with the NEBNext Ultra II FS DNA library prep kit. All genome sequencing was performed by the Pittsburgh Bacteriophage Institute and the libraries were run on an Illumina MiSeq instrument. Default parameters were used for all software unless otherwise specified. The read size, number of reads, and coverage for each genome are presented in Table 1. Raw reads were assembled with Newbler (v2.9) (2), yielding a single phage contig for each genome. The results were checked for completeness, accuracy, and genome termini using Consed (3). Sequence assemblies revealed the nature of the genome ends, and circularly permuted assemblies were bioinformatically linearized such that base 1 was assigned in accord with other microbacteriophages (4). Genomes were manually annotated with GeneMark (v2.5) (5), Glimmer (v3.02) (6) was used to assess start sites and coding potential, and Starterator (v1.2) (7) was used to summarize the starts across each Pham. To collect evidence for gene function and the validity of each gene product, HHpred (v3.2) (8), NCBI BLAST (9), the Conserved Domain Database (10), TMHMM (v2.0) (11), and SOSUI (12) were utilized. tRNAscan-SE (v2.0) (13) and ARAGORN (v1.2.41) (14) were utilized to identify putative tRNAs and transfer-messenger RNAs (tmRNAs). Once completed, the annotation was submitted to GenBank, and the data for each phage were also archived in Phamerator (15) and the Actinobacteriophage Database online at PhagesDB.org (7).

**TABLE 1:** Akoni, Ashton, and Truong characteristics and comparisons

Phage	GPS location	Location found	Genome size (bp)	GC content (%)	Total predicted genes	Read length (bp)	No. of reads	Shotgun coverage (×)	Type of genome ends	Nucleotide identity (%)		
										Akoni	Ashton	Truong
Akoni	28.124411 N 82.368309 W	Tampa, FL	54,307	60.1	55	150	225,866	261	Circularly permuted	100		
Ashton	28.06 N 82.41967 W	Tampa, FL	54,519	60.1	56	150	382,496	46	Circularly permuted	98.67	100	
Truong	29.831944 N 81.268889 W	St. Augustine Beach, FL	54,309	60.1	55	150	81,308	212	Circularly permuted	99.04	98.55	100

**FIG 1:**
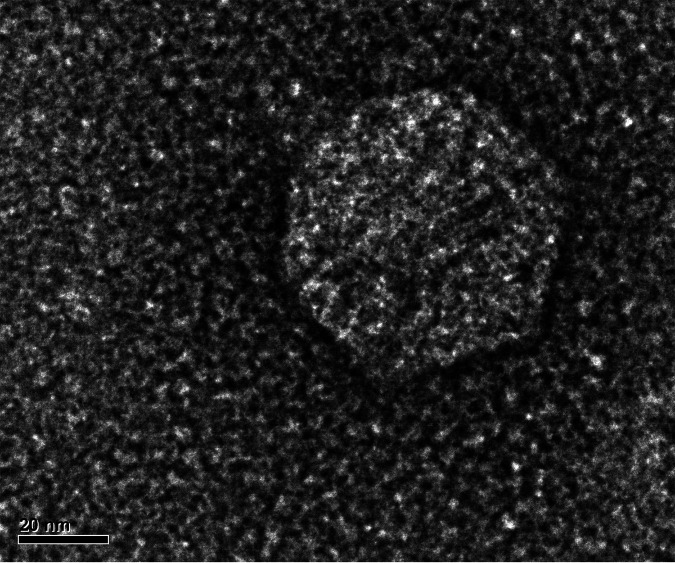
Transmission electron micrograph of *Microbacterium* phage Akoni. Phage lysates were negatively stained with 1% uranyl acetate. Both Ashton and Truong showed similar morphology (see https://phagesdb.org/phages/Ashton/ and https://phagesdb.org/phages/Truong). Scale bar = 20 nm.

Akoni, Ashton, and Truong all produce ∼1.5-mm clear plaques. Negative-staining transmission electron microscopy shows that Akoni has an icosahedral capsid of ∼60 nm and a short <10-nm tail (Fig. 1). The lack of defined tape measure genes validated the classification of these phage as *Podoviridae*. The three phages are in cluster EK2 (7, 15). The genomes of Akoni, Ashton, and Truong are 60% GC rich and have 55 or 56 predicted genes, of which only 13 could be assigned a putative function. Alignment of the genomes in Phamerator shows that Ashton has two small regions of nucleotide diversity that result in one additional predicted gene. Truong and Akoni show an average nucleotide identity (ANI) determined by OrthoANI (16) of 99.02%, while Akoni and Truong have ANIs of 99.00% and 98.92%, respectively, to Ashton (Table 1). A noteworthy feature of these EK2 phages is the presence of an ∼13,400-bp gene that encodes a 4,487-amino-acid protein of unknown function. This gene encompasses nearly 25% of the entire genome and is one of the largest ever found in the actinobacteriophages.

### Data availability.

This whole-genome shotgun project has been deposited in DDBJ/ENA/GenBank under the accession no. MK757449 and SRX11157091 for Akoni, MT310875 and SRX11157092 for Ashton, and MT310869 and SRX11157093 for Truong. The versions described in this paper are the first versions.
